# Obstetrics, and Diseases of Women and Children

**Published:** 1861-09

**Authors:** Wm. B. Atkinson

**Affiliations:** Lecturer on Obstetrics, and Diseases of Women and Children


### OBSTETRICS, AND DISEASES OF WOMEN AND CHILDREN.

BY WM. B. ATKINSON, M.D.,

LECTURER ON OBSTETRICS, AND DISEASES OF WOMEN AND CHILDREN.

### I.—*Treatment of Cases of Abortion in which the Membranes and Placenta are retained.* By DR. PRIESTLEY, London.

The dangers and morbid conditions arising from membranous and placental retention are—flooding; decomposition of the uterine contents, leading to local inflammation of the uterus and surrounding tissues, to phlebitis and phlegmasia dolens, and to general poisoning of the system, as evidenced by fever, peritonitis, rheumatic pains and abscesses; sub-involution of the uterus; and the generation of some of the forms of mole out of the tissue left in the uterus. The entire absorption of the placenta is uncertain.

The treatment by ergot, galvanism, or injection of cold water is very unreliable. The safest and best plan is the introduction of the fingers into the uterus, and the removal of the contents of that organ. Dr. Priestley had never known this to be followed by evil results. Of course, rough and careless manipulation is to be avoided: less risk is incurred by this method than by the retention of the placenta. It is important to effect the removal before putrescence, or contraction of the os. In six hours after the escape of the embryo, the placenta may be removed. The patient should be placed on her back, the thighs flexed on the abdomen, and while, with one hand, the operator steadies and depresses the uterus, the other is passed into the vagina, the os gently dilated with the index finger and the second next passed, if required. These form the best and most sensitive forceps. Chloroform may be administered if necessary; if fever, etc., be present as in cases of delay, the operation is not advisable. Dilatation may be effected by sponge-tents when necessary.—(*Med. Times and Gazette*, May 18, 1861.)

II.—*A new Instrument for the Reduction of Prolapsed Umbilical Funis.* By J. G. WILSON, M.D., Glasgow.

The instrument is a French invention, the inventor's name not stated. It consists of two finely polished whalebone rods, one of which is straight, with a handle of the same material, the other terminates in a hook, and slides upon its fellow by means of silver rings. The movement of the one upon the other is limited by means of a stop to about an inch, to prevent their separation. The hook is intended for the funis, and by the approach of the other rod is temporarily converted into a ring; length, including handle, 18¼ inches. The rods are as thick as the tubes of Gooch's canula. The prolapsed cord is grasped and carried up beyond the presenting part, and then released by separating the rods. Its advantages are simplicity of construction and easy application. In two cases in which Dr. Wilson employed the instrument, he succeeded perfectly in carrying up the funis; nor was it again prolapsed.—(*Glasgow Med. Jour.*, April, 1861.)

III.—*The Most Efficient Method of Removing the Placenta.* By DR. CREDE.

Dr. Crede insists on the removal of the placenta by uterine action alone, and objects to the introduction of the hand within the genitalia except in rare cases. His method consists in excitation and increasing of uterine activity; contraction is to be induced by friction and irritation of the fundus and body of the uterus, with the hand over the abdomen. It should be performed at once after the birth of the child. The friction at first should be gentle, then gradually increased. The author has in very many instances succeeded in a quarter or half an hour in inducing the necessary contraction, even where uterine action was ever so slight. When the contraction is strong, he grasps the fundus in the hollow of his hand, while the fingers make gentle pressure; no inconvenience results to the patient except the increased pain of this forcible contraction, which is more than compensated for by its rendering unnecessary the introduction of the hand within the sensitive parts. The womb remains well contracted, hemorrhage is less likely to occur, and inversion cannot take place.—(*Monatsschrift für Geburtskunde und Frauenkrankheiten*, April, 1860; *Amer. Med. Monthly*, June, 1861.)

IV.—*Inflammation of the Breast.* By Thos. Wm. Nunn, F.R.C.S., London.

Of 72 cases which fell under the observation of Dr. Nunn, 58 occurred during lactation, 7 during pregnancy, and 7 in women neither pregnant nor lactating. Of the 58, 56 per cent. occurred during the first two months of lactation; during the subsequent seven months only 14 per cent., and 29 per cent. after the ninth month. Over-lactation produced a cachexia, marked by a proneness to inflammation, dryness, and chalkiness of the skin, drowsiness, constipation, loss of appetite, and physical and mental lethargy. He found either breast equally liable, and the lower lobes twice as often affected as the upper.

Concerning the treatment, he thought continuous poulticing was decidedly mischievous, and hindered recovery, except where deep sympathetic pain and hyperæsthesia of the surface existed, when warm poultices are soothing. The recumbent position is of the first importance. In Dr. Nunn's hands, belladonna had not given encouraging results, though he could not doubt that in the earliest stage of congestion and as a preventive, it had a beneficial influence. The moment of opening the abscess should be vigilantly watched for, as the rapidity of cure depended upon this. He strongly advocated the use of galvanism of a low form of intensity, for the treatment of sinus and painful œdema after the acute symptoms have subsided.

Dr. Braxton Hicks had found equal parts of extract of belladonna and glycerin, applied externally, of great value in checking the secretion, at the same time giving internally iodide of potassium in 8-grain doses.

Dr. Tyler Smith cured cracks in the nipples by painting them with a strong solution of nitrate of silver. Abscess could often be prevented by belladonna, smearing the breast with equal parts of the extract and water. Bromide of potassium internally, and as a lotion, seemed to have a specific effect in diminishing the mammary secretion.

Dr. Tanner had great faith in belladonna, which he thought was not employed with sufficient freedom. The best plan was to paint the whole gland, and then apply a cold bread and water poultice twice or thrice a day.

Mr. Gervis regarded the delay in putting the child to the breast after delivery as a not unfrequent cause of acute mammitis. The child is not allowed to suck till the third day, and then the breasts are swollen and tender, the nipple retracted, and in drawing it out, the manipulation predisposes to inflammation.

Dr. Madge thought it not always necessary to wean the child in such cases, as by strapping the affected side all irritation and inflammation frequently subside quickly, and the mother is enabled to continue with the other breast.

Dr. Gibb thought that changes in the milk in prolonged lactation often caused abscess. The chief of these changes is a fermentation of the sugar at the moment of secretion from constitutional causes, giving rise to the formation of vibriones and monads, producing great irritation and a tendency to suppuration.

Dr. Richards thought nine-tenths of the cases were caused by sore nipples, which by their pain cause the mother to desist from suckling, resulting in engorgement, etc. When suppuration occurs, hot fomentations are best, and an early evacuation of pus. Local depletion and belladonna are worse than useless.

Mr. Owen had found great benefit, in sore nipples, from a lotion of borax, oil of almonds and water.—(*Lancet*, June 22, 1861.)

V.—*Abortion; Cold a Principal Cause.* By DR. GEORGE J. BENNET, N. Y.

Cold, as a cause of miscarriage, has been overlooked, though an ordinary cold, with the attending derangement of health, more frequently than any other cause produces this occurrence. Congestion of the pelvic viscera results, more especially when the uterus is in a pregnant state, separation of the placenta, hemorrhage, external or internal, follow, and, consequently, fœtal death.

In threatened miscarriage we must restrain the flow of blood, whatever the cause. If the cause be cold, with derangement of digestion, we find mild laxatives and alteratives best, as syrup of rhubarb and castor oil, āā f℥ss, cream of tartar, blue pill, etc., combined with opiates. Ipecacuanha, with nitrate of potassa, where febrile excitement exists, is a valuable anti-hemorrhagic, antispasmodic, and equalizer of the circulation.—(*American Medical Monthly*, May, 1861.)

VI.—*Puerperal Peritonitis treated with Opium.* By G. P. CADY, M.D., Nichols, Tioga County, N. Y.

C., aged 35, was delivered of her fifth child after a severe labor. On the third day she was attacked with peritonitis; had during the night several chills; her skin was dry and hot, countenance anxious, tongue furred, respiration hurried, pulse 140; bowels very tender and painful, with tympanitis, and sickness of stomach. Gave her four grains of opium at nine, and again at twelve o'clock, and then two grains every two hours for four hours, when she was thoroughly narcotized. She lay quiet, her skin was moist and itching, eyes suffused, pupils much contracted, respiration ten per minute, and sighing. She had no pain, but was easily roused. For four days complete narcotism was maintained, sometimes by two grains every hour, sometimes every two hours. On the third day she vomited several times, but the medicine was repeated oftener, and it subsided. On the fifth day the pulse began to fall; on the sixth, it was 85, and from that time she took four grains each of opium and quinia daily, for several days, when her appetite returned, and both were omitted. Result, a complete cure. She took thirty-four grains the first twenty-four hours, and then twenty to twenty-five per day.—(*American Medical Times*, Aug. 3, 1861.)

VII.—*Phlegmasia Dolens cured by Collodion.* By DR. R. LATOUR, Bordeaux.

Mad. ———, aged 20, was attacked about eleven days after delivery with phlegmasia dolens in the left leg, the swelling of which, by the third day, had extended from the upper portion of the thigh down to the ankle.

Dr. Latour attempted to prevent its extension upward by the application of collodion to the part. Immediately the progress of the inflammation ceased, and by the next day its intensity had much moderated. The coating was carried over the whole affected part, with the exception of the foot, which continued to swell. This also was covered, with a good result. By the seventh day after the institution of this plan of treatment, the parts had returned to their normal condition, and she was able to walk about as usual.—(*Jour. de Méd. de Bordeaux*, Jan. 1861.)

VIII.—*Case of Ovariotomy.* By P. S. Mootoosawmy Moodelly, G. M. M. C., Manargoody, Tanjore.

C., aged 24, admitted February 16, 1859, with a tumor on her left side. Had been tapped several times. Married ten years; had one child; menstruation ceased for last twenty-eight months. Swelling came on gradually, with occasional pain and febrile disturbance. As it increased, the pressure produced nausea and vomiting, oppression of respiration, and œdema of the legs. The abdomen now measures forty-four inches in circumference. Fluctuation is distinct in the tumor. General health much impaired. Tapping relieved her of twenty-six pints of a thick viscid brownish fluid, which coagulated on the application of heat. The fluid having reaccumulated, the operation of extirpation was performed March 6th. After the usual preparation, chloroform was freely administered, and an incision was made an inch and a half long in the median line, about an inch and a half below the umbilicus. No adhesions were found, except to the omentum at the upper portion. The incision was carried down three inches, and the tumor was tapped, discharging a thick, viscid, dark-brownish fluid, of the consistence of syrup, to the amount of several pints. The tumor now appeared thick and solid. The wound was slightly enlarged, and the mass drawn out without much difficulty. It was attached by a large neck to the broad ligament of the uterus. A double ligature was passed through it, and tied on each side, after which the mass was removed about half an inch below the point of ligation, no hemorrhage ensuing. The right ovary was healthy. The ligatures were allowed to recede into the abdomen, and the wound was closed by sutures and straps.

The after-treatment was conducted according to the emergency of the presenting symptoms. The ligatures were withdrawn on the forty-fourth day, and she left the hospital on the fifty-fourth, perfectly well. Fifteen months after the operation she remained well, the catamenia having returned. The tumor, which measured thirty-two inches in circumference, consisted of a thick sac, and several small cysts on its margin. The whole weighed twelve pounds and thirteen ounces, and contained twenty-one pints of fluid like that withdrawn.—(*Madras Quart. Jour. Med. Sci.*, July, 1860.)

IX.—*Ovarian Dropsy; Rupture of the Cyst internally; Cure.* By Dr. Milner Barry, London.

Occasionally, in cases of ovarian dropsy, rupture of the cyst occurs, which is generally followed by death, either from collapse or peritonitis; sometimes by a refilling of the cyst, rarely by a perfect cure.

H. A., aged 32, had ovarian dropsy for two years. She was as large as a female at the eighth month of pregnancy. She was treated for some weeks, and then left the infirmary without any apparent change. Some six months after, she tripped, and fell violently forward on a brick floor. For some days she was ill, but every day became smaller, and in three weeks had returned to her normal size. She could not recollect any increased action of the bowels or bladder at the time. Her catamenia are regular, and she has had no return of the dropsy, now some four years since.—(*Medical Times and Gazette*, July 13, 1861.)

X.—*Enlarged Ovary prolapsed into the Recto-Vaginal Cul-de-sac, and obstructing Labor; Rupture of the Uterus.* By DR. J. C. HUTCHINSON, N.Y.

The patient, aged 35, was in labor with her second child, the pains having commenced on the previous day. The os was well dilated, the membranes protruding, the vertex presenting, and the pains severe. A tumor was detected between the rectum and vagina, about the size and shape of the half of a large apple. In consequence of the pressure of the head above, the tumor could not be moved. An opiate was given, and sleep procured. At 12 M. the next day the forceps were applied, but without effect. In the evening vomiting commenced, the pains had subsided, and death resulted by the next morning.

The post-mortem examination revealed a rupture of the uterus, which was well contracted, the child being in the abdominal cavity. The tumor was the right ovary enlarged, containing bone, hair, and a soft matter like stearine. A bony tumor projected posteriorly from the body of the pelvis, to the distance of three-fourths of an inch. All the pelvic diameters were diminished.—(*American Medical Monthly*, July, 1861.)

XI.—*Uterine Hœmatocele.* By HENRY MADGE, M.D., London.

DR. MADGE read a paper before the Obstetrical Society upon this subject, in which he related the case of a lady who, having suffered from local peritonitis, went out too soon, was attacked with a profuse menstrual discharge, and was brought home in a state of collapse. When reaction came on, peritoneal symptoms were evident in the lower part of the abdomen, and a large tumor was found extending from the umbilicus into the pelvis; as found by rectal and vaginal examinations, the uterus was retroverted; the severe symptoms subsided, and about a month later, a discharge of blood took place per rectum, seeming to empty only the pelvic portion of the tumor. When the monthly period arrived, the discharge lasted two days, and on the second day, anæmia, partial collapse, and pain came on. It was evident that internal hemorrhage had again occurred. She rallied, when phlegmasia dolens attacked the left, and a week after, the right leg. When the next menstrual period arrived, there was a profuse discharge of clots and altered blood per rectum, for two days, to the amount of three or four pints; the abdominal tumor disappeared, and death ensued from prostration.

The post-mortem revealed a cavity in the pelvis, containing clotted blood, with bands of torn false membrane. The capsule of the left ovary was distended to the size of an orange, and divided into two cells by a band, each cell containing blood and clots. A small portion of the ovary remained. The right ovary was congested and fixed by adhesions. The case was one of "intra-peritoneal uterine hæmatocele," produced by a common cause, bleeding from ovarian vessels at menstruation.

DR. TYLER SMITH considered the affection to be essentially a form of ovarian or Fallopian menstruation, vicarious in character. Thus the blood accumulated, and by lymph being thrown out by the peritoneum, the sac is formed. The escape was by ulceration through the rectum. He had seen three cases in which this had occurred which resulted in cure. He would be strongly inclined to puncture the tumor through the rectum, and give emmenagogues to produce healthy menstruation.—(*Lancet*, March, 3, 1861.)

2.—*Ibid.* By DR. JACKSON, Boston.

DR. JACKSON reported to the Boston Medical Society a case where death occurred in a few hours, from a discharge of blood into the abdominal cavity. The Fallopian tubes were injected, and the left one ruptured, through which opening the blood had evidently passed, as a clot hung from the aperture. The patient was every day expecting her menstrual discharge.

3.—*Ibid.* By DR. GAY, Boston.

The patient had for some years experienced trouble at her menstrual periods. Four weeks ago, the discharge was suddenly checked by her taking cold. She was seized with intense bearing-down pains and great soreness, both of which steadily increased. She could not walk or stand straight, and was generally obliged to lie upon her right side. A vaginal and rectal examination revealed a painful swelling near the uterus and extending toward the sacrum. The patient lost flesh rapidly, and became very feeble; there were only short intervals of ease from pain. A general fullness of the abdomen was seen below the umbilicus. The case was diagnosed as *retro-uterine hœmatocele*, and an incision an inch long was made in the deep portion of the vagina. Large quantities of coagulated blood flowed out, and the discharge continued for several days. Two weeks after the operation and six weeks from the attack, her catamenia appeared, lasting two days, not profuse or painful.

She is now daily improving, with scarcely any vaginal discharge, and the swelling has entirely subsided. Many fetid "grayish pieces of flesh, like *torn rags*," were discharged.—(*Boston Med. and Surg. Jour.*, May, 1861.)

XII.—*Fungosities of the Uterus.* By DR. GOLDSCHMIDT, Strasburg.

The fungosities are formed by a simple hypertrophy of the elements which enter into the normal composition of the uterine mucous membrane. The elements which are transformed are the cells, fibres, glands, and capillary vessels. The disease may be only a hyperæmia, but more frequently the hypertrophy continues until soft, pulpy excrescences are produced, which are easily removed by an instrument or the finger-nail. They vary in size from that of a millet-seed to that of an egg. Occasionally a pedicle is formed, and they resemble strongly a polypus. The disease appears at all ages, but generally between 20 and 30. The predisposing influences are tedious labors and abortions. The principal and almost constant symptoms of this affection are hemorrhages and pains. Among their complications, the most important are softening of the uterine tissue, and suppurative inflammation of the ovaries. The hemorrhages generally become serious, causing great anæmia. Conception is hindered and abortion produced by these fungi. Cauterization and injections are useless; abrasion must be practiced with the *curette* of Recamier, or the finger-nail. The latter is fully as useful and less dangerous than the former; hence the curette should only be used in those cases where the former method is impracticable, as from the impossibility of passing the finger into the cavity. In some instances, cauterization with the nitrate of silver becomes an adjuvant, by modifying the surface thus scraped; scarcely an accident can occur from the operation, and the result in a vast majority of cases is entirely satisfactory; the hemorrhage and pains cease immediately, the uterus properly performs its functions, menstruation is regular, and conception again becomes possible.—(*Gaz. Med. de Strasbourg; Gaz Med. de Paris*, Mai 4, 1861.)

XIII.—*Amenorrhœa; its Treatment.* By J. Y. SIMPSON, M.D., etc., Edinburgh.

The symptomatic treatment of amenorrhœa will be that proper to restore the system to its normal standard, as the relief of the *asthma* which sometimes though rarely accompanies it. The readiest is the inhalation of chloroform, by breathing a few drops from the palm of the hand, using it more freely as may be requisite. We may also use carbonic acid gas, developed in a beer bottle, from a mixture of equal parts of crystallized bicarbonate of soda and tartaric acid, with about a tumblerful of water, and inhaled through an india-rubber tube through an opening in the cork. Leeches to the top of the sternum are useful when the health will permit, or dry cups in other cases, over the chest, before and behind.

*Neuralgia.*—Here the narcotics may be used, but still better, the solution of the arsenite of potassa, two drops thrice a day. If a rheumatic taint is present, thirty or forty drops of the tincture of actæa racemosa three or four times a day, in a little water, will be beneficial. Locally, apply sedative and anæsthetic liniments, or inject subcutaneously a watery solution of morphia. The neuralgic headache is often speedily relieved by heat to the forehead, as by a sponge squeezed out of hot water, or by mustard or turpentine. When chronic, admirable results have been obtained from nickel, in the form of the sulphate or phosphate, in doses of half a grain or a grain three times a day.

*Eruptions.*—Acne, which is wont to appear each month, when persistent may be attacked with the oil or butter of antimony, applied with a brush, and neutralized speedily with bicarbonate of soda. If it lose its effect, applications should be made alternately of citrine or other mercurial ointment, and of some of the preparations of iodine. The latter remedy stains the skin, so that many refuse its use, and hence a preparation of Dr. Arkwright, of Accrington, is best. This is composed of one part of tincture of iodine and two parts of the milder aqua ammonia. This mixture, which is at first brown, after standing forty or fifty hours becomes quite clear and colorless, and may be applied to the skin two or three times a day for a long time without producing pain or excoriation. Locally, Rumex ointment, or, if the eruption is very irritable, bismuth or cucumber ointment, with a drachm of officinal prussic acid added to each ounce. Internally, small doses of arsenic are often advisable.

The *constitutional* treatment, of course, would be according to the indications—deplete the plethoric, and give tonics to those below par. Especial attention must be given, in cases of dyspepsia, not to impose upon a stomach incapable of digestion. The most serviceable tonics are iron, manganese, zinc, arsenic, especially the phosphates of the first three.

*Specific* treatment will consist in the use of *emmenagogues*, of which the best is iron. Care should be taken not to give this remedy in doses sufficient to overload the digestive canal and impede its functions, which is the great mistake in the employment of iron.

*External applications,* as cupping-glasses, poultices, and various irritants, have been applied to the mammæ, or over the sacrum, or leeches to the anus in plethoric patients. The feet may be placed in hot water for four or five nights every month, or the hip-bath may be employed.

*Injections into the Rectum and Vagina.*—These are with a view of producing an excitement of the pelvic viscera, and thus causing a flow to the parts. The injections may consist of aloes, etc., thrown into the rectum, or aqua ammonia, f℥j, in a cup of milk, thrown into the vagina. The latter is regarded by the Italians as one of the most certain measures for restoring the catamenial flow.

*Emmenagogues applied to the Uterus.*—Nitrate of silver to the cervix has been found of service by the French. But the best means will be to apply direct stimulation to the uterus or uterine cavity, as the nitrate of silver, cantharides, or iodine. These may be applied by the porte-caustique of Lallemand, of sufficient length, and curved so as to adapt it to the uterine inclination. When charged, it is introduced into the interior of the uterus, about the menstrual period, and rotated twice or thrice. This has produced menstruation in a few hours.

*Dry-cupping* the interior of the uterus may be employed by means of a tube like a male catheter, pierced with thickly-set small orifices, for about two inches from its extremity, and having an exhausting syringe at the lower end. This, however, does not act purely as a dry-cup, for it is frequently found to fill with blood after being exhausted. Generally, several days are required before this effect is obtained.

Where the ovaries require stimulation, it is better to use the *galvanic intra-uterine pessary.* This is a small copper bulb, from the middle of which rises a stem, made half of copper and half of zinc, about two and one-third inches in length. The half next the bulb is copper, and the other is zinc. The stem is introduced within the cavity of the uterus, and there is on the other side of the bulb an opening, in which a staff may be fixed, with which the instrument may be carried up into its place. A gutta-percha pessary may be placed below it, to prevent it slipping. The whole should be removed occasionally, and the zinc cleansed from the white crust which soon accumulates upon it. This pessary has never produced any bad results, and has frequently caused an immediate improvement in all the symptoms. "*Probably other kinds of foreign bodies lodged there would produce much the same effect; and I have treated cases of amenorrhœa with success by the introduction of a stem-pessary made entirely of copper or of German silver.*"

*Electricity* and *galvanism* have also been found of value; but to apply them a special form of conductor is needed. When the affection is connected with an *under-sized uterus,* the galvanic pessary becomes most useful by developing the organism. Where the atrophy is great, of course this means will fail.—(*Med. Times and Gaz.,* May 18, June 15, 1861.)

2.—*Ibid.* By DR. ALTHAUS, London.

In young and unmarried women, Dr. Althaus has found *electricity* a valuable emmenagogue. Much depends on a proper knowledge of how to use the remedy, as well as perseverance in its employment. "As to the parts of the body to which the current should be directed, I may add that Faradization of the skin of the soles of the feet by means of wire brushes, or of the abdominal parietes by moistened conductors, and finally the application of one conductor to the nape of the neck, and of another over the os pubis, are the most effectual methods in cases of this kind."—(*Med. Times and Gaz.*, June 22, 1861.)

XIV.—*Hysteria treated by Veratrum Viride.* By Dr. G. M. STAPLES, Dubuque, Iowa.

In the treatment of this affection, Dr. Staples regards as an important indication the controlling of the circulation by cardiac and arterial sedatives. He believes that no other remedy is comparable with the veratrum viride for this purpose.

Mrs. P., aged 25, of highly nervous temperament, during her catamenial period, four months ago, received a fright, which occasioned *suppressio mensium* and convulsions. The attacks continued, sometimes daily, sometimes but once a week. The menstrual discharge returned in six weeks, and remained normal. In one attack the convulsions lasted sixty hours, almost without cessation. She had been treated by intelligent physicians with the usual remedies, but without avail, and her general system began to suffer.

Dr. Staples placed her upon the use of the fluid extract of veratrum, six drops every two hours, and she was continued under its influence. After the second dose the disturbance disappeared, but reappeared upon the discontinuance of the remedy. It was renewed, and continued three weeks, with the effect of producing a permanent cure.

In consequence of tenderness between the scapulæ, counter-irritation along the spine was also employed.

During the treatment violent catharsis was produced by the remedy, after the action of a cathartic. This would appear to militate against the opinion expressed by Dr. Otterson in the March number of the *American Medical Monthly*, who says that "veratrum is not cathartic at all."—(*Med. and Surg. Rep.*, May 18, 1861.)

XV.—*Irritation of the Urethra in Females.* By JOHN HUNTER, M.A., Manchester.

Having encountered several cases not particularly described in the books, Dr. Hunter gives a sketch of three cases, occurring in a female of advanced age, in one of middle age, and in a young woman.

Mrs. C., aged 72, for fourteen years suffered with incontinence of urine, and a constant desire to void the urine, but only with an aggravation of her sufferings. The external parts appeared and felt natural, but the urethra, from the meatus up, was very red and congested. On the introduction of a catheter, the whole urethra was found to be exquisitely tender. The bladder did not seem to be affected. The urine was not more acid than natural. The treatment consisted in the use of alteratives and small doses of liquor potassæ. Locally, a little warm water was injected to wash away the adherent mucus, taking care that the nozzle of the syringe was fully introduced, and that it did not impinge on the walls of the urethra. After this a syringeful of nitrate of silver, five grains to the ounce, was thrown up, and allowed to come away itself. This was repeated daily, and gradually the irritability became less, and the urine could be longer retained. The cure was complete and permanent in a fortnight.

M., aged 50, had suffered in a similar manner for four years. She was compelled to get up many times in the night to relieve the bladder. The urine was much clouded with mucus. The treatment, as above, did not do more than alleviate in this case; hence the following plan was adopted: After the daily injection, the patient was turned, so that her feet rested on the bolster of the bed; the pelvis was raised by means of pillows, and the shoulders depressed, so as to throw the urine away from the neck of the bladder. She was then instructed to hold the urine as long as possible, and when the desire could no longer be resisted, the urethra was previously anointed, by introducing an uncut quill smeared with the dilute nitrate of mercury ointment. This plan, by preventing the irritating urine from touching the walls of the urethra, allowed time for the passage to be healed, and recovery soon followed.

G., aged 22, had suffered from childhood. The same treatment was adopted, with the addition of an injection of a little sweet oil after the nitrate of silver. After she had much improved, an attack of gastritis caused her death.—(*Lancet*, May 18, 1861.)

XVI.—*Infantile Erysipelas.* By J. LEWIS SMITH, M.D., of New York.

This affection being rare among infants, a full knowledge can only be acquired by the aggregate experience of practitioners. In forming the tables, Dr. Smith rejects all attacks of umbilical erysipelas. He reports in detail 36 cases, of which 19 died; 24 were under six months, 9 from six to twelve months, and only 3 above one year.

Of 49 cases, the point of commencement was 13 times in the vulva; 16 in the arm from vaccination; 6, the leg; 2, near the ear; 1, the elbow; 1, the shoulder; 1, the chin; 1, the nates. Of 33 cases, 14 were males, 19 females. The causes are impure air, uncleanliness, and defective alimentation. In a large proportion there is generally an exciting cause, such as vaccination. This may arise from the cut or vesicle, but the virus itself may also be in fault, as some cases undoubtedly indicate.

In very young infants the disease may be traced to the mother, who is also attacked. He relates the following remarkable case:—

About the year 1840, Dr. Folsom, of New Bedford, Massachusetts, saw a case of a man with a pain in the knee, who died suddenly with obscure symptoms in two days. Dr. Elijah Colby, at the autopsy, pricked his finger, and not feeling any inconvenience, attended a case of labor. In a few hours he was taken sick, and left the case in charge of Dr. Folsom. She died in three days with puerperal fever. The infant, in two days, was seized with erysipelas, and died in one day. Dr. Colby's finger became swollen and painful, the lymphatics of the arm inflamed, and the axillary glands suppurated. There was, however, no appearance of erysipelas. In two weeks he resumed practice, and attended fifteen obstetrical cases. All the mothers died of metro-peritonitis, and the infants of erysipelas in a week after birth. The doctor temporarily retired.

The symptoms of infantile erysipelas are restlessness, pain, want of sleep, fever, the rash, not peculiar, though sometimes vesicated. There is a strong tendency for the disease to return.

The prognosis is very unfavorable. Under six weeks death generally ensues; up to six months recovery is doubtful; above that the majority recover. It is more favorable when the limbs are affected; when it spreads slowly; when superficial.

The duration averages fifteen days in cases which recover, and nearly ten in those that die. Death generally occurs from exhaustion.

The treatment in this country seems uniformly to be with the tinc. ferri muriat., beef-tea, and wine-whey. Across the Atlantic depletion is more generally employed; hence Bouchut's aphorism, "*the erysipelas of infants is a fatal disease.*"

The largest dose was ten drops every two hours to a child twenty months old, who recovered. Quinine was occasionally given as a tonic. Complications were treated as in other cases.

Local treatment to limit the disease is rather injurious than otherwise. The usual remedies may be employed to moderate the intensity of the inflammation. —(*Amer. Med. Times*, June 22, 1861.)

XVII.—*Treatment of Hooping-cough.* By C. S. SHELTON, M.D., Springfield, Illinois.

The treatment of pertussis by the use of the extract of belladonna and sulphate of zinc, as recommended by Dr. Fuller, in the *Lancet*, has been successfully tested by Dr. Shelton in sixteen cases. In each case beneficial results speedily followed, and in the greater number the cough was cut short, in many in about ten days. The ages of the children ranged from six months to ten years. Those under three years took, at the commencement, one-sixth of a grain of belladonna and half a grain of zinc four times a day, dissolved in mucilage. Above that age, the dose was a quarter of a grain of the extract and a grain of the zinc, increasing the dose, in some to double the quantity, according as the child could bear it. The speedy modification of the symptoms rendered it unnecessary in some, while others could not bear it. An hour after taking the medicine there resulted a deep-red color of the whole surface, particularly of the face and neck, dilatation of the pupils, arterial excitement, dry, warm skin, and general excitability of the system, all passing off in from one to three hours. The paroxysms would soon be less frequent and protracted, the hoop less loud, the spasm greatly subdued, less bronchial irritation, and the stomach less irritable. No other medicines were given in conjunction with these remedies.

One child of two years, though very ill, was cured in ten days; and in a second attack, from careless exposure to the cold, a like result followed. A boy of seven years, though the disease was gradually increasing in violence, in about ten days was perfectly well.

Dr. Shelton is led to believe that these remedies "possess peculiar power in reaching the seat of the disease, and that the hooping-cough need not run its course."—(*Amer. Med. Times*, July 20, 1861.)

2.—*New Remedy for Hooping-cough.* By M. Foster, F.R.C.S., Huntingdon, England.

Dr. Foster has, in about fifty cases, used the *trifolium in fœno*, or common clover hay, which failed only in three or four. It cures mostly in a few days, sometimes extending to a week or two. It relieves the spasmodic character of the paroxysms, while other symptoms are met with emetics, aperients, tonics, and salines. Since using it, Dr. Foster disbelieves in "the natural course" of pertussis. The remedy answers best when it acts slightly on the bowels.

The hay should be sweet and leafy. For the infusion :—℞. Trifolii in fœno, ℥ij; Aquæ bullient, Oj. Macerate for four hours and strain. For a child of five years, give a tablespoonful three times a day. The syrup :—℞. Trifol. in fœno, ℥ijss; Sacch. cand., ℥ij; Aq. bull., Oj. Macerate in the water for an hour with gentle heat, then boil down to proper consistence. Dose, two teaspoonfuls four times a day.—(*Med. Times and Gaz.*, May 25, 1861.)

